# Immunoblotting Identification of Diagnostic Antigens of *Paragonimus westermani* Type 1 for the Detection of Human Pulmonary Paragonimiasis in North East India

**DOI:** 10.3390/tropicalmed9010006

**Published:** 2023-12-22

**Authors:** Kangjam Rekha Devi, Archana Deka, Debdutta Mukherjee, Harpreet Kaur, Kanwar Narain

**Affiliations:** 1 Indian Council of Medical Research-Regional Medical Research Centre, Dibrugarh 786001, Assam, India; krekha75@yahoo.co.in (K.R.D.); archanadeka001@gmail.com (A.D.);; 2 Indian Council of Medical Research-Headquarters, New Delhi 110029, India; kaurh.hq@icmr.gov.in

**Keywords:** *Paragonimus westermani*, helminths, immunoblot, paragonimiasis, diagnostic antigens, immunological assays, host–parasite interaction

## Abstract

Human pulmonary paragonimiasis, an emerging concern in North East India, frequently masquerades as pulmonary tuberculosis due to clinical and radiological similarities, leading to diagnostic challenges. This research aimed to harness the immunoblotting technique to discern immunodiagnostic protein antigens from both adult worm and excretory–secretory (ES) extracts of the prevalent *Paragonimus westermani* type 1 in Arunachal Pradesh, North East India. We studied the time kinetics of immunoreactive patterns in relation to the duration of infection in rodent models. Immunoblot analyses were also conducted using sera from ELISA-positive patients confirmed with paragonimiasis, facilitating the selection of antigenic extracts with diagnostic potential. Further, ES protein antigens were subjected to 2D immunoblot analysis and immunoreactive protein spots identified using MALDI-TOF MS. The immunoreactivity patterns of ES antigens with sera of paragonimiasis-positive patients were detailed, and specific immunoreactive protein antigens were pinpointed using peptide mass fingerprinting (MALDI-TOF). This work underscores the enhanced diagnostic accuracy when combining ELISA with immunoblotting for pulmonary paragonimiasis in regions like North East India, marked by co-existing helminth infections.

## 1. Introduction

Paragonimiasis, caused by trematodes of the genus *Paragonimus*, is a zoonotic disease posing a significant health threat, primarily affecting economically disadvantaged communities in Asia, Africa, and Latin America where freshwater crab consumption is common [1,2,3]. Considered a Neglected Tropical Disease (NTD) by the World Health Organization (WHO), paragonimiasis is alarmingly prevalent, with an estimated 20 million infections globally [4,5]. The South Asian region, including countries like China, the Philippines, and Vietnam, reports the highest incidence, although this likely represents only a fraction of the actual cases [6,7,8]. Therefore, understanding the disease’s epidemiology, transmission pathways, diagnostics, and treatment efficacy is crucial.

There are over 40 species of *Paragonimus* worldwide, and 16 of these are pathogenic to humans [1]. Noteworthy among them are *P. westermani* in Southeast Asia, *P. africanus* in West Africa, *P. mexicanus* in South America, and *P. kellicotti* in North America [9]. The first intermediate host for *P. westermani* is snails, while freshwater crabs, shrimps, and crayfish serve as second intermediate hosts. The definite hosts of *Paragonimus* include human, cats, and dogs. The human pulmonary paragonimiasis disease cycle begins with the consumption of inadequately cooked freshwater crabs, which harbor the larval form of the parasite known as metacercariae [10]. The metacercariae penetrate the gut, excyst their larvae in the small intestine, and migrate through the diaphragm to the pleural cavity of the lungs. In the lungs, cysts are formed, where larvae mature into adult worms and start releasing eggs. The eggs are discharged in the sputum and can be detected in feces. The symptoms of paragonimiasis may include chronic cough, hemoptysis, respiratory or gastrointestinal issues, fever, anemia, and weight loss. Although the infection can be effectively treated using praziquantel, the clinical symptoms of the disease can be misleading (often resembling tuberculosis or lung cancer), making diagnosis complex [5,11,12,13].

In the Indian subcontinent, the first case of paragonimiasis was identified in Manipur in 1981 [14]. Subsequent investigations have pinpointed other states in the northeastern (NE) regions of India as hotspots for this disease, with *P. westermani* and *P. heterotremus* as the predominant lung fluke species [8,15,16,17,18]. Molecular diversity suggests genetic diversity within the *P. westermani* species, potentially indicating a species complex. Notably, a study by Devi et al. [19] identified two distinct genotypes of *P. westermani* from NE Indian freshwater crab, with one genotype (type 1) differing from its East Asian counterparts. This diversity can impact the disease’s behavior, underscoring the need for a nuanced understanding of host–parasite interactions, especially in terms of the antibody response [20]. For the development of an immunodiagnostic test, the use of locally available species of lung fluke, *Paragonimus westermani* type 1, appears to be an important source of parasitic antigens because it can easily be procured locally and developed in rodent experimental models.

During their life cycle in the definite host, lung flukes produce a vast array of components, including excretory–secretory products, tegumental proteins, cyst wall proteins, cystic fluid proteins, egg-derived proteins, etc. [10]. These diverse sets of proteins are essential for parasite survival and involved in pathogenicity. Excretory–secretory (ES) products are mixture of molecules (proteins, glycans, and lipids) released by lung flukesinto the host’s biological fluids. These secreted and excreted molecules are presented at the host–parasite interface and elicit an immune response in the host; they are therefore considered a good source for antigens with serodiagnostic potential [21]. Apart from excreted–secreted proteins, adult worms contain many proteins that are important in performing various functions, including nutrition invasion, host immune response, and immune evasion [22]. The use of adult worm protein in serological assays has demonstrated high sensitivity and specificity in the detection of positive cases of human paragonimiasis [23]. Different antigenic extracts from helminth worms have their own specific protein components and the interaction mechanisms of these molecules with the host immune system are dissimilar. Advancements in proteomics tools have facilitated analysis of the proteins expressed during the different development stages of helminth parasites. Moreover, an immunoproteomics approach based on the integration of 1D or 2DE immunoblot analysis with proteomic techniques has been successful in the characterization of specific immunogenic proteins recognized by infected individuals and elucidating new protein biomarkers for the development of new immunoassays [24].

ELISA based on *Paragonimus* antigenic extracts are highly sensitive serological tests but are not free from the issues caused by cross-reactive antigens, which may yield false positive results [25]. Hence, ELISA should be combined with immunoblotting assays, which will help in the early and accurate diagnosis of the disease. Given the prevalence of pulmonary paragonimiasis in India’s NE regions, particularly in Manipur, Nagaland, Arunachal Pradesh, and Assam, our study focuses on investigating the antibody response to different antigenic extracts of adult lung flukes. In this study, we conducted immunoblot analyses using the sera from parasitologically confirmed paragonimiasis patients (ELISA-positive) to identify antigenic extracts with diagnostic value. Further, the ES antigenic proteins released by adult lung flukes were analyzed using 2DE immunoblotting coupled with mass spectrometry to identify the immunogenic proteins recognized in infected sera.

## 2. Materials and Methods

### 2.1. Biological Samples

The metacercariae of *P. westermani* type 1 were harvested from freshwater crabs (*Maydelliathelphusalugubris*) collected from Arunachal Pradesh, NE India, as detailed in previous work [26]. Identification of the metacercariae in the dissected crabs was carried out using a stereomicroscope. The metacercariae were mainly isolated from the hepatobiliary pancreas and muscles of the infected crabs. The isolated *Paragonimus westermani* type 1 metacercariae were cleaned in 1X PBS and identified under a light microscope before the infection of Wistar rats. Protein extraction from a pool of *P. westermani* metacercariae was performed in microcentrifuge tubes containing protein lysis buffer (8M urea) added with 1X Complete Protease Inhibitor Cocktail (Roche, Mannheim, Germany), and homogenization carried out using sonication in ice (30 s, times) and an ultrasonic probe. The lysate obtained was centrifuged at 16,000× *g* for 20 min at 20 °C to pellet the cell debris. After centrifugation, the supernatant was transferred into a clean tube and stored in aliquots at −80 °C.

Female Wistar rats (approximately 2 months old, *n* = 50) were fed with 20 metacercariae, obtained from infected crabs [27]. The rats were dissected weekly (up to 15th week post-infection) and their blood was collected via cardiac puncture. After clotting at room temperature for 1 h, the blood was centrifuged at 1000–2000 g for 10 min at 4 °C to obtain the sera. The sera was stored at −20 °C for subsequent use. Adult *P. westermani* type1 flukes were harvested by sacrificing the infected rats after 45 days of infection. The lungs were dissected and washed in 1X PBS (pH 7.4), and the cysts were separated from the lungs to avoid rupture of the cysts. The cysts were again washed in 1X PBS and the worms inside the cysts were taken out, cleaned, and placed in 1X PBS. The use of the Wistar rats for pulmonary paragonimiasis was approved by the Institutional Animal Ethics Committee, ICMR-RMRC, Dibrugarh, India.

This study included human serum samples from selected freshwater crab-eating communities of Arunachal Pradesh, NE India, which were collected and tested in the previous study by the authors [28]. All subjects gave their informed consent for inclusion before they participated in the study. Serum samples from 22 parasitologically confirmed patients and the pooled sera of 10 healthy subjects were analyzed with immunoblotting. Confirmed patients exhibited symptoms such as chronic cough with hemoptysis, with negative sputum samples for acid-fast bacilli (AFB, *Mycobacterium tuberculosis*) but positive sputum samples for lung fluke eggs. The anti-Paragonimus IgG antibodies were confirmed using an in-house ELISA kit (the first indigenously developed test from India) [28]. The sample was considered positive when the absorbance values were higher than 0.5. Ethical approval was obtained from the Institutional Ethics Committee, ICMR-Regional Medical Research Centre, Dibrugarh, Assam, India. All the experiments were performed in accordance with the relevant guidelines and regulations.

### 2.2. Preparation of Adult Worm Somatic Antigens and Immunoblotting

Live adult *Paragonimus* worms were homogenized to obtain soluble somatic antigens [28]. Briefly, live or frozen adult worms were homogenized by employing the Roche MagNALyser instrument (Roche Applied Science, Penzberg, Germany) in cold 45 mm phosphate buffered saline (PBS) (pH 7.2) containing protease inhibitor cocktail solution (1X). The lysate obtained was incubated overnight at 4 °C. Next, the lysate was centrifuged at 15,000 g for 1 h at 4 °C to obtain an adult worm (AW) soluble somatic antigen. The samples were stored in aliquots at −80 °C. The protein concentration of the AW extract was estimated using the Bradford method [29] and the homogeneity of extract was determined by running the samples in Tris-Glycine SDS-PAGE gels [30]. Prior to gel electrophoresis, the protein sample was reduced by adding β-mercaptoethanol and heating it at 95 °C for 5 min. The protein bands in the gels were observed using Coomassie and silver staining procedures [31]. For the silver staining, the gel was incubated for 1 h in fixing solution containing methanol, acetic acid, and milliQ at a ratio of 1:5:4, respectively. Subsequently, the gel was washed with 50% ethanol solution, treated with 0.06% sodium thiosulphate pentahydrate solution, and incubated for 30 min with silver nitrate solution. After incubation, the gel was developed to visualize the protein bands

Immunoblotting was performed using sera from infected rats collected over time. At first, the AW protein sample was resolved in 15% Tris-Glycine SDS-PAGE gels procured from Bio-Rad (Hercules, CA, USA). The electrotransfer of proteins from the gel to the polyvinylidene difluoride membrane (PVDF) was performed in a Bio-Rad Trans-Blot transfer cell for 90 min at 100 V. Next, the blotted membrane was incubated with blocking buffer containing 5% BSA in wash buffer (0.1% Tween20 in Tris-NaCl; TBST) for 1 h. The blotted PVDF was cut into vertical strips and stored overnight with sera collected weekly from infected rats. The rat sera were diluted in the ratio 1:100 with blocking buffer. An uninfected rat serum was taken as negative control group. Next, the membrane strips were washed in TBST and incubated in a solution containing the secondary antibody (dilution ratio 1:2500) HRP-conjugated anti-rat IgG (Promega Corporation, Madison, WI, USA). The membrane strips were then washed with TBST and the antibody–antigen reaction was observed using the Promega TMB (3,3′,5,5′-tetramethylbenzidine)chromogenic substrate.

### 2.3. Preparation of Excretory–Secretory Antigenic Proteins

Live adult worms collected from experimental rats were used for the preparation of excretory–secretory (ES) antigenic proteins [28]. The adult worms were first washed at least 10 times in 1X PBS (pH 7.4) and then soaked in 1 mL of 1X PBS (pH 7.4) for 3 h under shaking conditions. The adult lung flukes were removed and the spent culture was centrifuged at 15,000× *g* to eliminate eggs and debris. The supernatant was treated with a complete Roche Protease Inhibitor Cocktail solution and filtered through a 0.2 µm membrane to obtain the ES proteins. The protein concentration of the ES antigens was determined using Bradford assay. Subsequently, the ReadyPrep™ 2-D clean-up kit (Bio-Rad Laboratories Inc., Hercules, CA) was used for the quantitative precipitation of the sample proteins and removal of contaminants [32]. The dried pellet obtained after cleanup was suspended by adding 125 µL of rehydration buffer and stored at −80 °C. To check the quality of the antigenic extract, two replicates of the reduced ES sample (5 µg each) were resolved in Tris-Glycine SDS-PAGE and stained using Coomassie Brilliant BlueR250. The Bio-Rad Precision Plus (catalog no. 161–0377) protein marker was used for determining the molecular weights of the protein bands.

### 2.4. Immunoblot Analysis Using Human Serum

Immunoblotting was performed using AW and ES protein antigens and the pooled sera of confirmed pulmonary paragonimiasis patients. The protein antigens were reduced using β-mercaptoethanol and loaded on to SDS-PAGE gels to perform gel electrophoresis using a Bio-Rad Mini-PROTEAN Tetra cell (Hercules, CA, USA). After the completion of electrophoresis, a portion of the gel was cut and subjected to silver staining. The remaining part of the gel was used for the immunoblotting procedure as described above. The blotted membrane was incubated overnight at 4 °C with the sera of confirmed pulmonary paragonimiasis patients (diluted 1:100 ratio). For the negative control, the pooled sera of 10 uninfected individuals were used. The strips were incubated with secondary antibody (dilution ratio 1:1000). Immunoreactive proteins were visualized using a chromogenic substrate.

The ES protein samples were also separated in two-dimensional gel electrophoresis (2D-PAGE). At first, isoelectric focusing was carried out in 7 cm (pH 3–10) IPG strips (Bio-Rad, Hercules, CA, USA) under standardized running conditions [33]. Isoelectric focusing was performed in the Bio-Rad PROTEAN IEF Cell at 50 µA per strip; Step 1: 250 V for 20 min, Step 2: 4000 V for 2 h; Step 3: increasing voltage from 4,000 V for 3–4 h until1 4000 V-h. After focusing, the strips were equilibrated in two equilibration steps—10 min in Equilibration Buffer I containing DTT, followed by 10 min incubation in Equilibration Buffer II containing iodoacetamide. The equilibrated strips were applied to 12% SDS-PAGE gels and electrophoresis carried out. The gel was stained using Coomassie Brilliant Blue to visualize the protein spots, and selected protein spots were subjected to in-gel trypsin digestion.

Briefly, the stained gel was first washed with miliQ water twice followed by the excision of protein spots of interest. The gel pieces were diced into small pieces and further destained using an ammonium bicarbonate (NH_4_HCO_3_) solution and acetonitrile (1:1 ratio). Next, the gel pieces were dehydrated using acetonitrile until completely dry. The gel particles were rehydrated in freshly prepared 10 mM dithiothreitol (DTT) in 100 mM ammonium bicarbonate solution and incubated for 1 h at 56 °C in water bath. After incubation, the DTT was removed and the gel pieces treated with freshly prepared 55 mM iodoacetamide (IAA) in 100 mM of NH_4_HCO_3_ solution for 45 min. The supernatant was removed and the gel pieces were incubated with NH_4_HCO_3_ solution for 10 min. After the gel pieces had shrunk, the supernatant was removed and the gel was again dehydrated with acetonitrile for 10 min and vacuum-centrifuged until completely dry. Subsequently, 10 µL of freshly prepared trypsin solution (10–20 ng/µL in NH_4_HCO_3_ solution) was added to tubes containing the gel pieces and kept for overnight incubation at 37 °C. The solution was collected using centrifugation and transferred into fresh microcentrifuge tubes. The extraction buffer (acetonitrile and 0.1% trifluoroacetic acid = 1:1 ratio) was added to the gel pieces and sonicated for 20 min at room temperature. The fresh supernatant obtained using centrifugation was mixed with the previous supernatant. The supernatant collected was treated using a Speedvac until completely dry. The dried pepmix was resuspended in a solution containing acetonitrile and 0.1% trifluoroacetic acid (1:2 ratio). The peptides obtained were mixed with an α-Cyano-4-hydroxycinnamic acid MALDI matrix and the resulting 2 µL was spotted onto the MALDI plate. After air-drying the sample, it was analyzed on the MALDI-TOF mass spectrometry instrument and further analysis was undertaken with flex analysis software for obtaining the peptide mass fingerprint. The masses generated in the peptide mass fingerprint were submitted to MASCOT for protein identification against the *Paragonimus* Uniprot database [34].

The remaining gels were used for immunoblot analysis as described above. To study the interactions of the protein spots with the serum antibody, the blotted PVDF membrane was incubated with the pooled sera of confirmed patients (diluted 1:100).

## 3. Results

### 3.1. Protein Profiling of Different Antigenic Extracts

**Figure 1:**
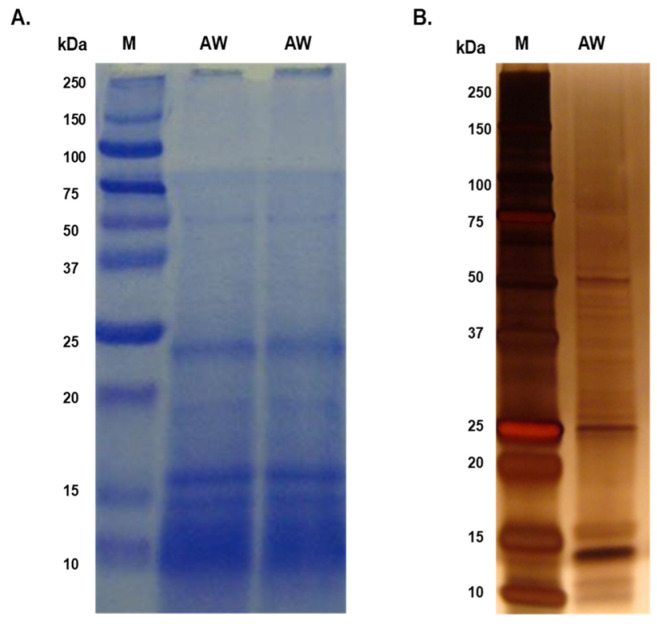
Separation of adult worm (AW) soluble somatic antigenic proteins using SDS-PAGE. (**A**) 10 µg of AW was resolved in 15% SDS-PAGE and stained with Coomassie Brilliant Blue; (**B**) 1 µg of AW resolved in 4–12% SDS-PAGE gel and silver-stained.

**Figure 2:**
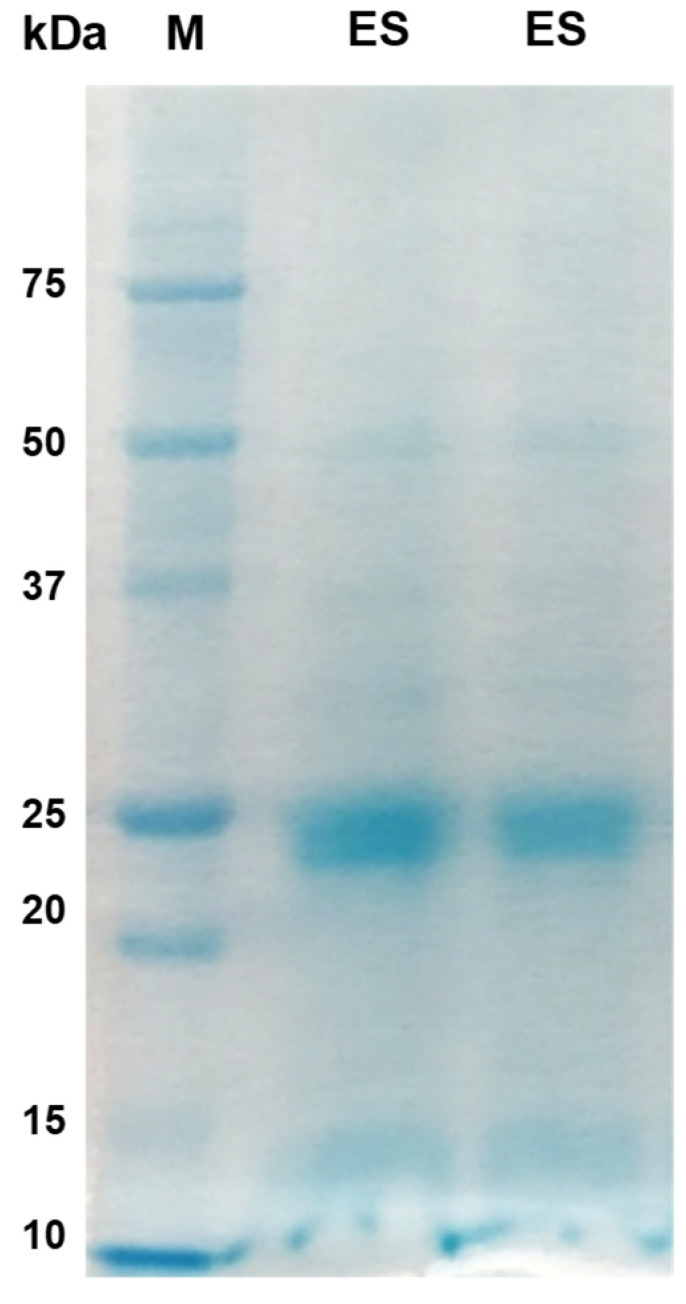
Excretory–secretory (ES) protein antigens of *P. westermani* type 1 resolved in 12% Tris-Glycine SDS-PAGE.

The majority of proteins in both the AW and ES extracts fell within the molecular mass range of 10 kDa to 50 kDa, with faint bands above 50 kDa in the AW extract (Figure 1; Figure 2). Prominent protein bands were observed in both extracts, with a cluster around 20–25 kDa and faints bands near 37 kDa observed in the SDS-PAGE gels.

### 3.2. Serum Antibody Responses in Experimental Rats

**Figure 3:**
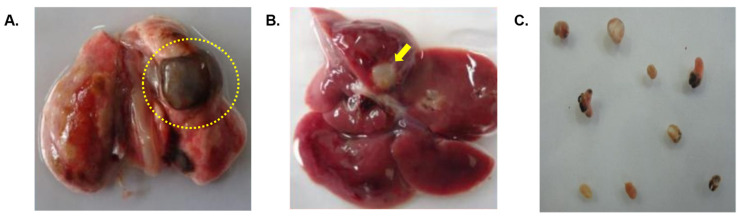
(**A**) Infected lung of a Wistar rat with cysts (dotted circle) containing adult *P. westermani* type 1 lung flukes. (**B**) Cysts formed in liver of Wistar rats due to *Paragonimus* infection indicated by arrow. (**C**) Adult lung fluke worms in 1X PBS.

**Figure 4:**
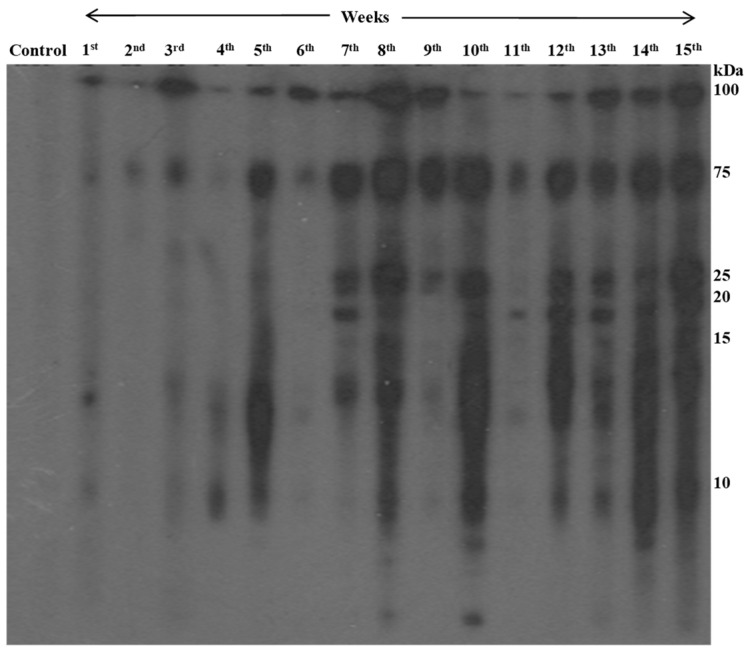
Chronological changes in antibody reactivity against adult worm antigens of *P. westermani* type 1 analyzed using immunoblotting.

The rats infected with metacercariae developed juvenile worms in the peritoneal cavity, and later, adult worms in the lung cysts, with parasitic cysts also found in the liver (Figure 3). From the third week onward, the lungs developed black cysts while after the seventh to eighth week, parasitic cysts were observed in the liver. Using immunoblotting, we monitored IgG antibody development against AW antigens (Figure 4 and Table 1). Reactive fractions emerged from the first week, expanding up to 15 weeks. Protein bands of molecular masses ~100, ~75, ~14, and ~9 kDa began to react with the experimental sera at the first week. Subsequently, the protein bands displayed increased reactivity up to the 15th week post-infection. Additional protein bands of molecular masses ~25, ~22, and ~19 kDa displayed immune reactivity from the seventh week post-infection. The pre-infection sera from experimental rats were non-reactive to the AW antigens.

### 3.3. Immunoreactivity of Human Sera with Antigenic Extracts

**Figure 5:**
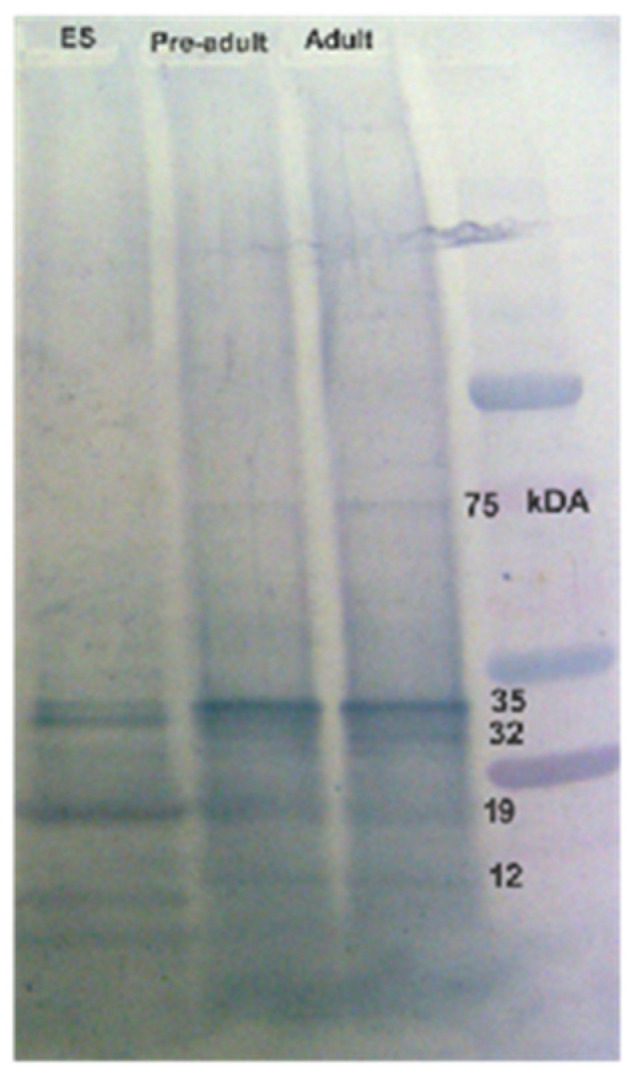
Immunoreactivity of human sera (pooled) from confirmed paragonimiasis patients of NE India against lung fluke antigens (*P. westermani* type 1).

The serum antibodies from confirmed paragonimiasis patients reacted with specific protein bands, including ~34 and ~25 kDa of the adult worm antigens (Figure 5). In addition, the infected human sera displayed slightly weak immunoreactivity against ~75 and ~15 kDa AW protein antigens. The protein antigens of the *P. westermani* metacercariae (pre-adult stage) were also reactive with the infected sera. The reactivity profiles of the metacercarial proteins and AW proteins were found to be similar. Two major protein bands (~34 and ~25 kDa) of the ES antigens showed strong immunoreactivity with the pooled lung-fluke-infected human sera.

**Figure 6:**
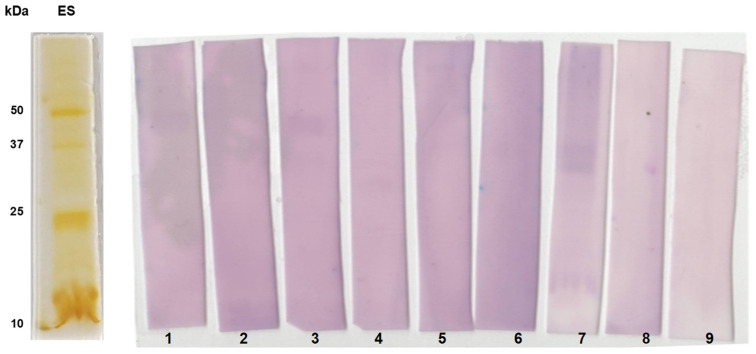
1D immunoblot analysis using excretory–secretory protein antigens of *P. westermani* type 1. Silver-stained gel showing banding patterns of ES antigen. Blotted membranes were incubated with individual human serum of ELISA-positive samples (Lanes 1–7) and known negative control samples (Lane 8 and 9).

Further, we studied the immunoreactive patterns of the individual serum of confirmed paragonimiasis patients (*n* = 7) with ES proteins using immunoblotting (Figure 6). Particularly, the protein bands of size 25 and 35 kDa were found to be highly immunoreactive.

### 3.4. 2D Immunoblot Analysis of ES Antigens

**Figure 7:**
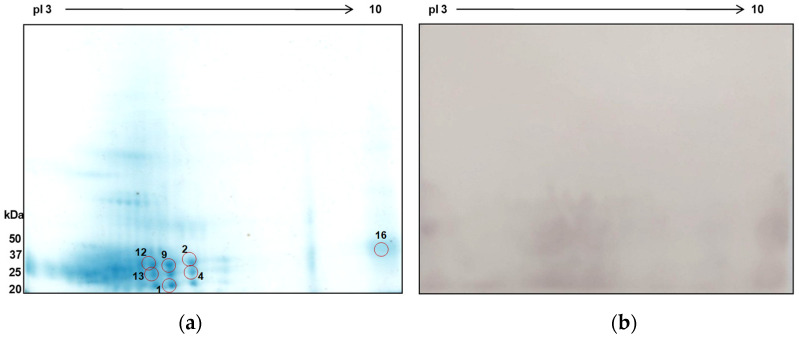
2D-PAGE of total ES protein antigens of *P. westermani* type 1 and corresponding immunoblot. (**a**) 2D gel profile of ES protein extract. Red circle indicates the protein spots positively identified by mass spectrometry. (**b**) 2D immunoblot profile using the pooled sera of paragonimiasis ELISA-positive cases.

Numerous protein spots were identified within molecular weights and pI ranges of 25 to 50 kDa and pI 3–6, respectively (Figure 7A). Seven protein spots were positively identified using mass spectrometry, with matches predominantly from *P. westermani* and some from other related species (Table 2). Seven peptides of protein spot 1 were found to match with the globin family profile domain-containing protein of P. westermani upon MASCOT search. Cathepsin F, a cysteine protease, was identified in spot 9. The other proteins identified in this study were carboxylesterase B, nucleosome assembly protein 1-like 1, 5′-Nucleotidase C-terminal domain protein, Ras and EF-hand domain-containing protein, and SET domain-containing protein. These proteins were previously not reported from ES extracts of *P. westermani*. Furthermore, the ES antigenic extracts were tested against the sera of confirmed paragonimiasis patients. The major immunoreactive clusters were found in the molecular mass region of 25–50 kDa and pI range 4–8. The MS-identified proteins were effectively recognized by the host humoral immune response and therefore determined to be antigenic with serodiagnostic potential.

## 4. Discussion

Human pulmonary paragonimiasis disease is a pressing public health concern in the northeastern (NE) states of India, primarily caused by *Paragonimus* species such as *P. westermani* and *P. heterotremus*. Notably, the NE Indian form of *P. westermani* type 1 has distinct characteristics from its East Asian counterparts [26]. While prior studies have reported on the immunodominant antigens of different antigenic extracts of lung flukes [34,35], the protein profile and immunoreactivity patterns of *P. westermani* type 1 of NE Indian origin remain elusive. Understanding the protein composition of antigenic extracts is crucial, as it offers insights into the biologically active molecules involved in the infection’s pathophysiology. In this context, we investigated the protein profiles of adult worm (AW) somatic and excretory–secretory (ES) protein extracts of *P. westermani* type 1 using 1D SDS-PAGE. The presence of a doublet protein band at 21/23 kDa and a diffuse band at 35 kDa supports exposure to *Paragonimus* species. These bands are known to contain highly immunogenic antigens [22,34,36]. Notably, a ~15 kDa in the ES sample aligns with the previous identification of host-tissue-derived hemoglobin [34]. The presence of host proteins in the ES extracts could indicate symbiotic interactions, aiding the parasite’s survival and infection process [37].

Further, we aimed to unveil the pathology and dynamics of the antibody response following infection with *P. westermani* type 1. In order to infect definite hosts, metacercaria should excyst into the host intestine. The cyst wall of metacercariae contains proteins that protect the juveniles and help them to evade the host immune system [38,39]. The appearance of parasitic cysts in the experimental animals correlated with detectable antigen-specific antibodies. The antibody response varied based on the maturation stage of the lung flukes, suggesting a dynamic interaction. Similar observations were also reported previously [27].

Laboratory diagnosis of human pulmonary paragonimiasis typically relies on detecting parasite eggs in clinical samples [28,40]. However, this method is not always feasible, especially at early stages or extra-pulmonary infections [41,42,43]. In these cases, serological assays are valuable, particularly in regions where paragonimiasis coexists with other infections like tuberculosis. ELISA and immunoblot assay are common approaches to detecting parasite-specific antibodies [44,45,46,47]. Several studies have evaluated the serodiagnostic potential of adult worm somatic extracts, excretory–secretory antigens, and recombinant proteins of *Paragonimus* species using ELISA and Western blot for the screening of human paragonimiasis [28,35,36,48]. Taking into consideration the genetically and geographically diverse complex of *P. westermani* populations found in Asia [49,50,51], it is crucial to identify and characterize the repertoire of antigenic proteins present in different samples. During their life cycles in the host, the quantity and type of molecules secreted/excreted at different developmental stages by lung flukes may greatly vary. Also, the type of immune response triggered in the host and interaction of parasite-derived components with the host molecules depends on their stage of development. Therefore, the present study evaluated different antigenic extracts of *P. westermani* type 1 (pre-adult/juvenile, adult worms and excretory-secretory) for their potential as serodiagnostic antigens using the sera of parasitologically confirmed pulmonary paragonimiasis human patients. The results obtained in this study are in line with previous studies [35]. The immunoreactive antigens of adult *P. westermani* were identified to be cysteine proteases of molecular weights between 27 and 35 kDa that largely reacted with the sera of paragonimiasis patients [34]. Although the banding pattern varied among the samples, it could recognize the different proteins of molecular size 10–75 kDa. The developmental stage of parasites responsible for infection in patients could be one of the explanations for the reactivity pattern variation. The antigenic determinants of proteins are lost during the denaturation process in immunoblot assay, while the same protein in its native form may expose relevant epitopes in an ELISA test. Studies on parasitic diseases have suggested the use of Western blotting and ELISA as confirmatory tests for positive cases in order to distinguish false positive sera from true positive sera [52,53,54]. In summary, combining ELISA and immunoblotting could enhance the serodiagnosis of human pulmonary paragonimiasis in endemic areas.

Identification of *Paragonimus*-species-derived proteins has been possible due to advancements in proteomics, and such studies have provided valuable insights into the biology of parasites and the identification of drug targets. Moreover, proteomics-based studies on diverse parasite antigenic preparations has facilitated unveiling s repertoire of antigens involved in host–parasite interactions [31,55]. A recent study explored the proteome of extracellular vesicles in excretory–secretory products released by *P. kellicotti* adult worms, providing new insights into the biology of host–parasite interactions and its potential implications in the development of novel diagnostic methods [56]. Excretory–secretory proteins of helminth parasitic worms are known to be ideal candidates for serodiagnostic protein antigens due to host–parasite interactions [21]. A two-dimensional gel electrophoresis (2DE)-based immunoblotting approach combined with mass spectrometry is the most popular research technique to obtain an overview of the specific protein antigens recognized in human patient sera [57,58]. Hence, to gain insight into the proteins present, we analyzed the ES extracts of *P. westermani* type 1 worms using 2D-PAGE, followed by protein identification. Cathepsin F and globins were identified in this study. The high content of globins in adult lung flukes was reported previously, making it another diagnostic candidate, but it requires further exploration [21,22]. Cysteine proteases, which have implications in pathogenesis and immune modulation [21,49], are known to share high sequence similarity among many *Paragonimus* species. Selecting unique regions of these cysteine proteases and the use of recombinant technology might prove useful for designing species-specific diagnostic kits. Lately, the cathepsin F gene expression has been detected at different developmental stages of *P. westermani* worms, and the recombinant protein expressed was found to be highly immunoreactive with paragonimiasis patient sera [59]. This study supports cysteine proteases as promising diagnostic targets. The carboxylesterase B domain-containing protein identified in this study may have a role in the metabolism of parasitic helminths and might be associated with resistance against anthelmintics [60]. Overall, the findings in this study provide a foundation for identifying potential diagnostic antigens. However, more comprehensive proteomics studies using high-throughput technologies are warranted to fully characterize the proteins of various antigenic preparations involved in the host–parasite interaction. The identification of potential diagnostic immunogenic proteins may assist in the development of more specific diagnostic tools that may eliminate the requirement for crude antigenic extracts. In recent years, there has been growing research interest in understanding glycoproteins and glycolipids of parasitic helminths, which have the potential to contribute to the development of novel intervention tools for helminth diseases [61,62]. The highly immunogenic glycans are widely shared among helminths and often lead to high cross-reactivity. The glycosylated moieties of proteins can greatly influence their immunogenicity, and hence the elimination of glycan structures via periodate oxidation increases the selection of more specific candidates for diagnostic tests [63,64,65]. Thus, exploring the glycoprotein profile for different life stages of the *Paragomius* parasite using a mass-spectrometry-based glycomic approach requires immediate attention that may contribute to understanding the importance of glycobiology in parasite development and the role of glycans in host–parasite interaction.

## 5. Conclusions

Our study has successfully identified specific antigenic fractions with immunodiagnostic potential for paragonimiasis. By using an experimental infection model involving laboratory rats and sera from confirmed human paragonimiasis cases, we have pinpointed antigenic markers that hold promise for accurate diagnosis. Moreover, we have unraveled the temporal evolution of immunoreactivity in response to the duration of infection in rodent models of paragonimiasis. These findings are anticipated to significantly contribute to the diagnosis of chronic stages of paragonimiasis. Importantly, this investigation marks the first of its kind in India, shedding light on the reactivity patterns between *P. westermani* protein antigens and sera from ELISA-positive patients using immunoblotting. Our results underscore the value of combining ELISA and immunoblot assays to effectively screen for paragonimisis in endemic regions. Of particular significance is the identification of cathepsin F using MALDI-TOF MS analysis, representing a highly promising serodiagnostic antigen. The potential of cathepsin F, along with other antigenic candidates, merits further exploration for the development of diagnostic kits that offer enhanced sensitivity and specificity for paragonimiasis. Overall, this study contributes valuable insights to the realm of paragonimiasis diagnostics and provides a foundation for continued advancements in the field.

## Figures and Tables

**Table 1:** Immunoreactivity pattern of different protein bands at different points in time post-infection.

Experimental Groups	Immunoreactivity
Control groups	No immunoreactivity
1st week	Faintly reactive, detected at 100, 75, and 14 kDa
3rd week (corresponding with development of lung cysts)	Detected at 100 and 75 kDa
7th week (sexually mature adult worms)	Detected at 100, 75, 25, 20, and 14 kDa
8th week onward (up to 15th week)	More intense pattern, detected at 100, 75, 25, 20, 15, and 10 kDa

**Table 2:** Seven protein spots identified by MALDI-TOF mass spectrometry.

Sample ID	Protein Name	Species	Accession No.	Theoretical Mass/pI	Score	Coverage (%)	Peptides Matched	E-Value
Spot 1	Globin family profile domain-containing protein	*P. westermani*	A0A5J4NY79	16,259/5.23	68	50	7	0.0077
Spot 2	5′-Nucleotidase C-terminal domain-containing protein	*P. westermani*	A0A8T0D4N5	50,306/5.93	66	28	12	0.013
Spot 4	Carboxylesterase type B domain-containing protein	*P. westermani*	A0A8T0DIE9	37,545/5.35	58	19	9	0.079
Spot 9	Cathepsin F	*P. westermani*	A0A5J4N7R7	35,237/5.64	59	36	11	0.069
Spot 12	Nucleosome assembly protein 1-like 1	*P. skrjabini miyazakii*	A0A8S9YXX6	38,362/5.56	55	33	10	0.15
Spot 13	Ras and EF-hand domain-containing protein	*P. westermani*	A0A8T0DUJ8	25,151/6.74	60	42	9	0.044
Spot 16	SET domain-containing protein	*P. skrjabini miyazakii*	A0A8S9Z7N9	44,115/8.92	43	18	7	2.3

## Data Availability

Data are contained within the article.
